# Real-Time Projections of SARS-CoV-2 B.1.1.7 Variant in a University Setting, Texas, USA

**DOI:** 10.3201/eid2712.210652

**Published:** 2021-12

**Authors:** Kaitlyn E. Johnson, Spencer Woody, Michael Lachmann, Spencer J. Fox, Jessica Klima, Terrance S. Hines, Lauren Ancel Meyers

**Affiliations:** University of Texas at Austin Department of Integrative Biology, Austin, Texas, USA (K.E. Johnson, S. Woody, S.J. Fox, L. Ancel Meyers);; Santa Fe Institute, Santa Fe, New Mexico, USA (M. Lachmann);; University of Texas at Austin Office of the Vice President for Research, Austin (J. Klima);; University of Texas at Austin Department of Population Health, Austin (T.S. Hines)

**Keywords:** COVID-19, coronavirus disease, SARS-CoV-2, severe acute respiratory syndrome coronavirus 2, viruses, respiratory infections, zoonoses, testing, variant of concern, university, Texas, Alpha variant, B.1.1.7

## Abstract

We used the incidence of spike gene target failures identified during PCR testing to provide an early projection of the prevalence of severe acute respiratory syndrome coronavirus 2 variant B.1.1.7 in a university setting in Texas, USA, before sequencing results were available. Findings from a more recent evaluation validated those early projections.

Identification of the highly transmissible novel severe acute respiratory syndrome coronavirus 2 (SARS-CoV-2) variant B.1.1.7 (Alpha variant) in the United Kingdom raised concerns for renewed pandemic surges worldwide (*1*,*2*). B.1.1.7 likely arrived in the United States by October 2020 (*1*); it was first detected in December 2020 and declared the dominant strain in April 2021, as projected in January 2021 (*3*). However, the regional prevalence of B.1.1.7 was largely unknown in early 2021 because of limited molecular surveillance for SARS-CoV-2 (*4*). To provide local situational awareness at that pivotal moment in the coronavirus disease (COVID-19) pandemic, we estimated the prevalence of B.1.1.7 on the basis of 17,003 student SARS-CoV-2 PCR test results reported through the Proactive Community Testing program at the University of Texas (UT; Austin, Texas, USA), a large public university located in a metropolitan area with a population >2 million, during January 16–February 12, 2021 (K.E. Johnson et al., unpub. data, https://doi.org/10.1101/2021.03.05.21252541). Those early estimates were subsequently validated by using PCR data through April 9, 2021.

Mutations in the B.1.1.7 spike protein result in a failure to detect the spike gene probe in standard SARS-CoV-2 quantitative reverse transcription PCR (qRT-PCR). In estimating the prevalence of B.1.1.7 from local quantitative PCR data, we initially assumed US estimates for the proportion of spike gene target failures (SGTF) attributable to B.1.1.7 (*4*) and, in our retrospective analysis, update that proportion on the basis of local sequencing data. We used a Bayesian model to estimate the local growth rate of B.1.1.7 among all SARS-CoV-2 infections and applied a compartmental susceptible-exposed-infected-recovered model of SARS-CoV-2 transmission to project the effect of B.1.1.7 on future COVID-19 prevalence.

We previously estimated that the relative frequency of B.1.1.7 among positive SARS-CoV-2 samples was growing logistically at a daily rate of 0.077 (95% CI 0.017–0.140), corresponding to an early doubling time of 9.0 days (95% CI 5.0–41.0 days) (K.E. Johnson et al., unpub. data, https://doi.org/10.1101/2021.03.05.21252541). At the time, we projected that B.1.1.7 would comprise most cases at UT by March 5 (95% predictive interval [PI] February 20–March 28) (Figure, panel A).

Subsequent estimates of B.1.1.7 prevalence based on quantitative PCR data from February 20 through April 9 fell within 95% PIs of the early projections (Figure, panel A) but suggested a lower daily growth rate of 0.037 (95 CI 0.026–0.048) and a corresponding doubling time of 18.7 days (95% CI 14.3–26.7 days). As of April 9, we estimated that B.1.1.7 comprised 61.2% (95% CI 48.5%–72.6%) of SARS-CoV-2 infections, consistent with our initial projections that B.1.1.7 would become the dominant variant by March 28 (95% CI March 20–April 10) and that B.1.1.7 is 24% (95% CI 17%–32%) more transmissible than the wild-type virus.

**Figure:**
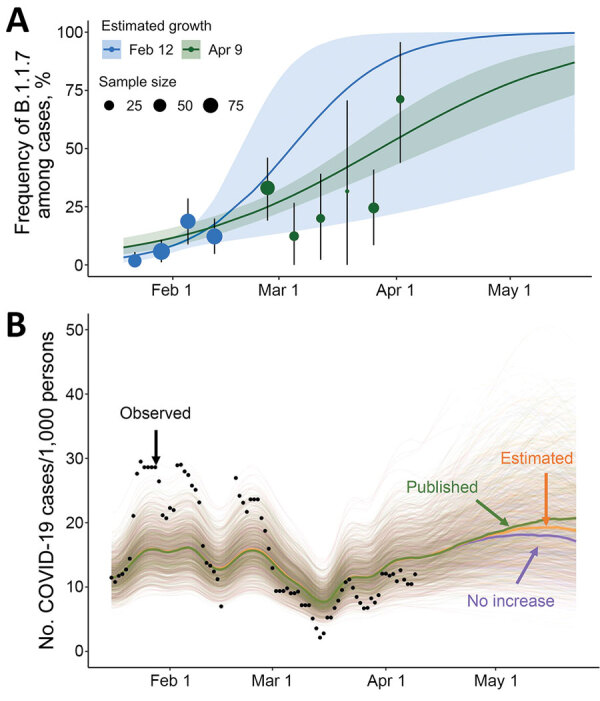
Estimated frequency of the B.1.1.7 variant among COVID-19 cases at the University of Texas and its projected impact on COVID-19 prevalence, Texas, USA, January 16–May 23, 2021. A) On the basis of the number of samples with spike gene target failures among severe acute respiratory syndrome coronavirus 2–positive samples reported by the University of Texas Proactive Community Testing Program (PCT), we estimated the weekly frequency of the B.1.1.7 variant (points); vertical error bars indicate 95% CIs. We fit a logistic growth model to data through February 12 (blue) and April 9 (green) to project the prevalence of the B.1.1.7 variant relative to the previously circulating wild-type virus through May 23. Shaded bands indicate 95% credible intervals, which reflect uncertainty in the percentage of cases that are spike gene dropouts, the percentage of spike gene dropouts that are B.1.1.7, and the fitted model parameters. The 95% credible interval of our initial projections (blue shading) contains the posterior median estimated from subsequent data (green line). B) Projected COVID-19 cases at the University of Texas through the end of the spring semester. Green, orange, and purple indicate projections with variant transmissibility from published literature, with the university-derived estimate, and with no transmissibility increase from the variant, respectively; black dots indicate the 7-day average reported positive cases per 1,000 persons detected through PCT. The projections assume a reproduction number (R_t_) of 1.17 (95% CI 0.94–1.43) as of April 9, on the basis of a recent estimate from PCT data (*5*,*6*). Spaghetti lines display 500 simulations; bold lines indicate the median projected value on each day. A lower-transmission scenario is described in the Appendix (https://wwwnc.cdc.gov/EID/article/27/12/21-0652-App1.pdf). COVID-19, coronavirus disease.

Based on those local estimates, scenario-based projections suggested that B.1.1.7 might cause 6.2% (95% PI 3.7%–8.4%) more cumulative infections during April 9–May 23, 2021, than if it were not more transmissible than the wild-type virus (Figure, panel B). When we assume a higher published estimate for the relative transmissibility of B.1.1.7 of 59% (95% CI 56%–63%) (2), we projected that B.1.1.7 would increase overall incidence by 14.3% (95% CI 10.8%–18.0%) during this period (Figure, panel B; Appendix Figure 5). We provide projections as total infections, rather than hospitalizations or deaths, because the primary concerns of the university at the time of this analysis were anticipating increased demand for isolation facilities, testing, and contact tracing. In either scenario, if behavior stays constant for the remainder of the semester, then we would not expect B.1.1.7 to drive a major surge in infections in the university community during this period (Figure, panel B). The relatively small effect derives from 2 factors that constrained future growth of B.1.1.7. We estimated that, by April 9, 47% (95% CI 39%–57%) of the student community was immunized by prior infection (either viral variant providing complete immunity) and that B.1.1.7 already comprised most (61.2%) new cases. This result hinges on the assumption that previous infection from either viral variant confers immunity to both variants and therefore would not apply to any type able to evade vaccine- or infection-acquired immunity. Our projections, which do not consider future behavioral change or reflect the full range of uncertainty, were not intended as forecasts but rather as plausible guideposts to help the university anticipate the severity of B.1.1.7.

UT surveillance testing indicates that B.1.1.7 rapidly became the dominant variant during the spring 2021 semester. Our methodology enabled rapid detection of B.1.1.7 emergence from widely available quantitative PCR data when sequence confirmation was not available or delayed, while quantifying uncertainty in the variant growth rate and fraction of SGTF samples that were positive for B.1.1.7. During January 16–March 5, UT confirmed 22 of 23 sequenced SGTF SARS-CoV-2 specimens as the B.1.1.7 variant, corroborating our reliance on SGTF data (Appendix).

Our findings reinforce the urgent need for expanded molecular surveillance capacity. In the absence of widespread and rapid sequencing efforts, quantitative PCR data from large-scale testing efforts have provided sentinel warning of B.1.1.7 emergence in cities throughout the United States.

Appendix Additional information about real-time projections of the SARS-CoV-2 B.1.1.7 variant in a university setting, Texas, USA.
