# Supplementary material for: Real-Time Projections of SARS-CoV-2 B.1.1.7 Variant in a University Setting, Texas, USA
Source: Emerg Infect Dis. 2021 Dec;27(12):3188–90. doi: 10.3201/eid2712.210652 (PMC8632165; doi:10.3201/eid2712.210652)
Supplement: Appendix — Additional information about real-time projections of the SARS-CoV-2 B.1.1.7 variant in a university setting, Texas, USA. [file 21-0652-Techapp-s1.pdf]

Article DOI: https://doi.org/10.3201/eid2712.210652

# Real-time Projections of SARS-CoV-2 B.1.1.7 Variant in a University Setting, Texas, USA

## Appendix

### qRT-PCR Data from University Testing Program

We analyzed de-identified lab results from the Proactive Community Testing (PCT) program at the University of Texas at Austin (*1*). Saliva specimens were collected from individuals at the university presenting for voluntary asymptomatic testing (*2*). PCT test results are based on the Thermo Fisher TaqPath COVID-19 Combo Kit, which targets three SARS-CoV-2 viral regions (N gene, S gene, and ORF1ab). Since samples are deidentified before analysis and some individuals may test more than once, there may be duplicate individuals in the data that could bias our estimates. Test results from positive cases, together with sample collection dates and reverse transcription quantitative polymerase chain reaction (RT-qPCR) cycle threshold (Ct) values for all gene targets were used to build the dataset. Ct refers to the number of cycles needed to amplify viral RNA to reach a detectable level. Ct values are inversely related to the amount of virus in a specimen.

Specimens are considered SARS-CoV-2 positive when at least two of the three target genes (N, Orf1ab, and S) are detected at a Ct value below 37. Following approaches from prior studies (*3*) (N.L. Washington et al., unpub. data, https://doi.org/10.1101/2021.02.06.21251159), we filtered our dataset for positive samples with strong amplification of the N gene (Ct <28) to increase the sensitivity and specificity of SGTF detection.

S gene target failures occur when RT-qPCR fails to detect the virus' S gene and are caused by mutations in the gene. Deletions in the amino acids H69 and V70 in the B.1.1.7 variant result in an SGTF. Samples were considered to be SGTF samples if they were positive for both N and Orf1ab, and negative for S. While SGTF can occur due to other mutations, the presence of the SGTF is one of several mutations that distinguish the B.1.1.7 variant from other

strains (*4*) (N.L. Washington, et al., unpub. data, https://doi.org/10.1101/2020.12.24.20248814). All of the SGTF specimens are sent for additional confirmation via sequencing through the university, but sequencing results are often delayed by one to 2 weeks. Given the need for rapid estimation of variant prevalence, we did not analyze the limited sequencing data that were available at the time of this study. Specifically, only four of the 31 SGTF identified as of February 12 had been sequenced.

Our analysis of B.1.1.7 variant prevalence focuses on the number of positive samples with SGTF out of the total number of high quality (Ct<28) positive SARS-CoV-2 samples collected through PCT. In the U.S., ≈70%–90% of SGTF samples were confirmed as variants in mid-January 2021 (N.L. Washington et al., unpub. data, https://doi.org/10.1101/2021.02.06.21251159). We used this national proportion in estimating the prevalence of B.1.1.7 based on the SGTF data. Our method includes a prior (β) distribution governing the proportion of SGTFs that are B.1.1.7. In the absence of sequencing data from UT, we originally set this before be relatively uninformative. We then updated it as we learned of the results from several SGTF specimens sequenced at UT. This distribution can continue to be updated as sequencing data on SGTF specimens becomes available.

We focused on the RT-qPCR data to estimate B.1.1.7 prevalence and growth rate relative to other strains. However, to provide situational awareness to the university on infection prevalence, we used all reported infections through the PCT program (regardless of their Ct value/viral load) to estimate the effective reproductive number ($R_t$).

To estimate $R_t$ from the reported PCT case data, we corrected for fluctuations in testing levels throughout the semester by calculating the PCT test positivity throughout the semester and using the imputed cases if the average number of tests conducted per day was uniform throughout the semester. On days without cases (this testing wasn't available on the weekends), we imputed the positivity using the 7-day rolling average of the PCT positivity. However, during the Texas winter storm, there was a 10-day pause in testing from February 13th to February 21, 2021, resulting in a (likely artificial) dip in the 7 day average. We corrected for this by taking the average test positivity from 3 days before and 3 days after the freeze and imputing the test positivity for the 10 missing days.

Using the median model estimate of the prevalence of B.1.1.7, we impute the amount of cases that are B.1.1.7 versus wildtype on each day of reported infections.

### Projecting B.1.1.7 Frequency Using a Logistic Growth Model

To estimate the relative frequency and growth of the B.1.1.7 variant, we implement a Bayesian logistic growth model using default priors in the rstanarm package in the R programming language (*5*). To start, let be the number of positive case samples with SGTF and low Ct, $B_t$ be the (unknown) number of B.1.1.7 cases at time $t$, and $N_t$ be the total number of positive case samples.

The goal is to estimate the prevalence of B.1.1.7, that is, the percentage of COVID+ cases which contain the variant at time $t$, which we denote by $p_{t,NB}$. Ideally, we would like to sequence the positive cases to detect B.1.1.7, in which case we would assume each COVID+ sample has a $p_{t,NB}$ probability of being B.1.1.7+, so then the number of B.1.1.7+ samples can be described by a binomial distribution

$$B_t \sim \text{Binomial}(N_t, p_{t,NB})$$

Previously, the growth in prevalence of the B.1.1.7 in other countries has closely followed a logistic curve (N.L. Washington et al., unpub. data, https://doi.org/10.1101/2021.02.06.21251159), so then the prevalence may be described to evolve over time given by the logistic equation

$$\log \frac{p_{t,NB}}{1 - p_{t,NB}} = c + kt$$

Here, $k$ is the growth rate and $c$ is an intercept term. These coefficients can be estimated using existing regression software implementations. However, the main problem is that we do not know the true number of B.1.1.7 samples. Instead we will impute this number using the number of SGTF samples, and the proportion of SGTF samples $p_{SB}$ that are estimated to be B.1.1.7. We describe uncertainty in the fraction of B.1.1.7 samples to total SGTF samples by a β distribution

$$p_{SB} \sim \text{Beta}(40, 10)$$

In our original estimates up through February 12th, the parameters of this β distribution were selected so that the 95% central credible interval is approximately (0.7, 0.9), consistent with the range of findings reported (N.L. Washington et al., unpub. data, https://doi.org/10.1101/2021.02.06.21251159) of percent of B.1.1.7 among SGTFs during mid-January 2021 in several U.S. states. As of this time, sequencing results of SGTF positive specimens from UT were not yet processed, and we therefore wanted to account for uncertainty in the percent of SGTFs that were B.1.1.7. By the time of our updated estimates through April 9th, we had learned that 22 out of 23 SGTF positive specimens sequenced by UT were confirmed to be the B.1.1.7 variant, resulting in a more informed β distribution of:

$$p_{SB} \sim \mathrm{Beta}(0.5 + N_{B.1.1.7}, 0.5 + N_{SGTF} - N_{B.1.1.7})$$

The 95% central credible interval for this distribution is (0.814 - 0.995) with a median value of 0.949. We had also learned that a non-SGTF sample could be a B.1.1.7 sample due to weak sequence alignment in the S-gene. However, we did not have sequencing data available at this time to identify the proportion of non-SGTFs that were B.1.1.7. In absence of an a priori estimate, we estimated the proportion of non-SGTF samples $p_{nSB}$ that are B.1.1.7 as between 1 and 5% (using a uniform distribution).

We implement the logistic regression binomial sampling model for $B_t$ as described above, integrating over the uncertainty in $p_{SB}$ via Monte Carlo sampling. One Monte Carlo draw of this model works as follows

1. Draw from the β distribution described above for $p_{SB}$ the fraction of SGTFs that are positive for B.1.1.7 and draw from a uniform distribution for $p_{nSB}$ the fraction of non-SGTFs that are B.1.1.7
2. Impute B.1.1.7 cases by multiplying SGTF cases by the draw from the β distribution plus non-SGTFs by the draw from the uniform distribution
3. Estimate the logistic growth model using this set of imputed B.1.1.7 case numbers,
4. Finally, project future B.1.1.7 prevalence using the fitted model

We combine all draws for projected B.1.1.7 prevalence to integrate over uncertainty in the fraction of B.1.1.7 to S gene dropout samples.

## Estimating the Transmissibility Advantage of B.1.1.7 for Infection Projections

We make projections using two estimates for the transmission advantage of B.1.1.7 relative to the wildtype. The first is a published estimate for the U.S (*6*). To account for uncertainty in this estimate, we sample from a triangular distribution with a mode given by the median estimate, and minimum and maximums given by the bounds of the 95% confidence interval.

The second is a new estimate from UT's PCT data, based on the rate at which the relative frequency of B.1.1.7 ($p_{B.1.1.7}$) increases, as given by

$$k = \frac{d}{dt} log(\frac{p_{B.1.1.7}}{1 - p_{B.1.1.7}})$$

This measures the rate at which the B.1.1.7 variant would displace wildtype variants in terms of the increase in the log(odds) to encounter the B.1.1.7 variant. The relative growth rate $k$ is the growth rate parameter that results from fitting the proportion of B.1.1.7 over time to the logistic growth model described above. Because the ratio of relative frequencies is equal to the ratio of the absolute numbers of variant versus wildtype samples, we can write $k$ as (*6*,*7*):

$$k = \frac{dlog(B.1.1.7)}{dt} - \frac{dlog(WT)}{dt}$$

Where $WT$ refers to all non-B.1.1.7 samples. This tells us that the relative growth rate of B.1.1.7 over wildtype, $k$ is also equal to the difference in the growth rates between B.1.1.7 ( $r_{B.1.1.7}$) and wildtype ($r_{WT}$) (*6*,*7*)

$$k = r_{B.1.1.7} - r_{WT} = \Delta r$$

To get a dimensionless relative selection coefficient, we multiply the relative growth rate by the mean generation time, $T_G$:

$$s_T = kT_G$$

If we assume that the generation time of the B.1.1.7 variant remains unaltered (*6*), it is possible to relate the selection coefficient $s_T$ to the expected multiplicative increase in the infectiousness of the virus, as measured by the ratio of the basic reproduction number $R_t$ of B.1.1.7 relative to that of the wild type. Assuming the generation time is gamma distributed and

the standard deviation of the generation time relative to the mean is small, we can approximate $R_t$ as (*8*):

$$R_t \approx exp(kT_G)$$

From this, we can write the ratio of the effective reproductive number of B.1.1.7 ( $R_{B.1.1.7}$) relative to the wild type ($R_{WT}$), i.e., the expected multiplicative factor on the transmission rate $\beta$, $M$ as:

$$M = \frac{R_{B.1.1.7}}{R_{WT}} = \frac{exp(r_{B.1.1.7}T_G)}{exp(r_{WT}T_G)} = exp(\Delta r T_G) = exp(kT_G)$$

For example, using the median estimate of relative growth rate, $k = 0.037$ (based on the fit to the logistic model using PCR data through April 9, 2021) and a generation time, $T_G$ = 5.8 days (*9*) yield an $R_t$ multiplier $M$ = 1.24, which is lower than the published estimate of $M$ = 1.59 [95% CI 1.56–1.63] (*6*).

For the locally derived estimate, we incorporate uncertainty on the multiplicative factor on the transmission rate, *M*, and its effect on transmission rate in future projections (described below) by sampling from the posterior distributions of the logistic growth rate *k* and calculating the corresponding *M* for each simulation.

## Projections of COVID-19 Spread at UT, Spring 2021

We project the effect of the spread of the B.1.1.7 variant at UT through the remainder of the spring semester of 2021 using an SEIR epidemiologic model (Appendix Figure 2), described by the equations below. We assume that (i) the increase in transmissibility of the B.1.1.7 variant is directly related to its growth in relative abundance as described above (*6*), (ii) the proportion of cases caused by B.1.1.7 is 61.2% [95% CI 48.5 – 72.6%] in Austin on April 9, and (iii) the proportion of cases caused by B.1.1.7 grows according to a logistic curve until reaching 88.2% of all infections by the end of the semester (May 23, 2021). Individuals transition between the states: susceptible (S), exposed (E), infected (I), and recovered (R). The symbols S, E, I, and R denote the number of people in that state. The model equations are given by:

$$\frac{dS}{dt} = -\beta(t)\frac{SI}{N}$$

$$\frac{dE}{dt} = \beta(t)\frac{SI}{N} - \gamma E$$

$$\frac{dI}{dt} = \gamma E - \delta I$$

$$\frac{dR}{dt} = \delta I$$

where $\beta(t)$ is the time-dependent transmission rate, $\gamma$ is the exposed rate, and $\delta$ is the recovery rate. The time-dependent transmission rate, $\beta(t)$, before April 9 is based on observed test positivity (described below), and implicitly incorporates the B.1.1.7 variant. The model does not distinguish between asymptomatic and symptomatic infections and thus implicitly assumes that asymptomatic and symptomatic cases have the same transmissibility. In the focal college student population, symptomatic cases are often mild and do not self-isolate in time to prevent onward transmission.

To estimate the SARS-CoV-2 transmission rate before April 9th, $\beta_t$, we used the publicly available daily numbers of positive tests and total tests administered by PCT collected from January 16th to April 9th, 2021 (*1*). For this analysis, we did not exclude positive cases with high Ct values. To correct for fluctuations in testing levels, we calculate the "test-level corrected" case count as the total number of positive cases divided by the total number of tests administered, multiplied by the average number of tests per day administered between January 16th and April 9th. We estimate the daily reproduction number (Rt) using the EpiEstim package (*10*) applied to the test-level corrected case counts. We account for uncertainty in $R_t$ by sampling directly from the gamma distribution estimated on each day $R_t$ is estimated (*10*). The transmission rate ($\beta_t$) corresponding to each $R_t$ is given by:

$$\beta(t) = R_t \delta \frac{N}{S(t)}$$

We made projections for the spread of SARS-CoV-2 after April 9 under two transmission rate conditions and three variant transmissibility conditions (for a total of six scenarios). Each scenario assumes either a slower or faster transmission rate (both sampled from the observed distribution of $R_t$) and one of three possible values for the relative transmission rate of the B.1.1.7 variant. To model B.1.1.7 transmissibility, accounting for the fact that the observed $\beta_t$

implicitly accounts for the spread of the variant ($p_{B.1.1.7}$) through April 9 ($t'$), we assume B.1.1.7 has a multiplicative impact on $\beta$ as given by

$$f(t) = \frac{p_{WT}(t) + Mp_{B.1.1.7}(t)}{p_{WT}(t = t') + Mp_{B.1.1.7}(t = t')}$$

where $p_{WT}(t) = 1 - p_{B.1.1.7}(t)$. We simplify this as

$$f(t) = \frac{1 + (M - 1)p_{B.1.1.7}(t)}{1 + (M - 1)p_{B.1.1.7}(t = t)}$$

We use the calibrated logistic growth model to draw matched samples from the posterior estimate of the logistic growth rate $k$ and the current prevalence of B.1.1.7, $p_{B.1.1.7}(t = t')$ to calculate $f_t$. The resulting $\beta(t)$ is given by

$$\beta(t) = f(t)\beta_s$$

where $\beta_s$ is chosen based on the scenario of interest (growing or declining epidemic in absence of the variant). The model assumes that infection by either variant renders one immune to infection by the other variant, which is consistent with reported estimates of immune evasion/reinfection from B.1.1.7 (*6*,*11*). The initial conditions are given in Appendix Table 2, the model parameters are given in Appendix Table 3, and the scenario parameters are given in Appendix Table 4.

## Initial Conditions

For each simulation, we sample from the distribution of proportion immune (previously infected) and the distribution of the proportion arriving to campus infected. The initial number of infected students is estimated using the observed number of positives out of the total number of tests administered to students over the first 4 days of testing (January 16th - January 19th). If we assume each individual has a probability $p_{t=0,inf}$ of being infected, the number of observed positives, $N_+$, out of the number of tests administered,$N_{tests}$, can be described by a binomial distribution:

$$N_+ \sim Binomial(N_{tests}, p_{t=0,inf})$$

If we assume a flat β prior on the probability of being infected at t = 0 of $p_{t=0,inf} \sim Beta(1,1)$, we can write the posterior probability of a student being infected as:

$$p_{t=0,inf} \sim Beta(1 - N_+, 1 + N_{tests} - N_+)$$

Using the number of positives and the total number of tests from the first week, we draw from the posterior distribution of the probability of a student being infected, then impute the initial number infected by multiplying by the total number of students at UT.

The assumed distribution of proportion immune is based on (i) estimates for the number of cases in August based on reported COVID-19 incidence in the residential counties of 30,000 returning students (*12*) and (ii) $R_t$ values from August 20th to January 16th, 2021 estimated from all student positive cases using the EpiEstim package (*10*). Of note, when estimating $R_t$ from total student cases for the Fall 2020 semester, we observed outliers on days that UT tested thousands of students planning to attend UT football games (September 2, 3, and 11). The average number of tests per day on those dates was 1,100 compared to 350 on other dates (*1*). The football testing data were irreversibly aggregated with proactive testing data. Thus, to avoid bias, we replaced the reported positives on those 3 days by averaging the six nearest days of reliable data. For 9/2 we averaged the values from 8/29 through 9/6 (excluding 9/1–9/2); for 9/3, we averaged 8/30 through 9/7 (excluding 9/1–9/2); for 9/11, we averaged 9/6 to 9/14 (excluding 9/11).

Using the SEIR model and workflow presented above, the daily transmission rate $\beta$ was calculated directly from $R_t$. The total number of cumulative infections for each simulation was used to estimate the initial number of individuals in the recovered compartment at the start of the spring semester. We assumed 5% of the student body was recovered and immune before the fall semester, and we assumed that all students previously infected were immune to either the wildtype or B.1.1.7 variant. If prior immunity was much higher than our estimates, then our projections would overestimate prevalence throughout the spring. If immunity is incomplete, allowing for some level of reinfection by the wildtype or B.1.1.7 variant, then our projections may underestimate the potential surge. $\beta(t)$ is either held constant after April 9th, or multiplied by $f(t)$ to simulate the increased transmissibility of the B.1.1.7 variant.

## Projected Infections under Each Scenario

The projections suggest that the rapid emergence of the B.1.1.7 variant is unlikely to lead to much higher COVID-19 prevalence in the UT community through the duration of the spring semester under any of the scenarios chosen (Appendix Figure 6). To evaluate the potential effect of the B.1.1.7 variant on transmission in the university setting, we ran paired simulations—one assuming elevated B.1.1.7 transmissibility and the other assuming baseline transmissibility. For each matched pair, the two runs assumed identical values for the transmission rate, initial infection prevalence, and starting B.1.1.7 prevalence. One run then assumed a specified higher transmissibility for B.1.1.7 (either based on (*6*) or our local estimates) and the other assumed no increase. After running the simulations, we computed the percent increase in cumulative infections during the period from April 9th to May 23rd, 2021 compared to the cumulative infections without the variant present. We performed these simulations for a "faster spread" scenario, assuming a constant transmission rate given by an $R_t>1$ pulled from the $R_t$ previously estimated on February 2nd (red line below), and a "slower spread" scenario, assuming a constant transmission rate given by an $R_t<1$ pulled from the $R_t$ previously estimated on January 29th (blue line below). We chose previously estimated $R_t$ values to obtain realistic uncertainty in the $R_t$ when we made projections (Appendix Figure 6). We note these represent scenario-based projections assuming constant behavior, not forecasts/predictions of what is expected to happen in the future due to the many other factors that contribute to transmission that were not directly modeled here.

In the worst-case scenario (faster baseline transmission combined with the published estimate of ~59% higher B.1.1.7 transmissibility), we find that B.1.1.7 would be expected to cause 475 [95% PI 238–825] more infections between April 9 and May 23, 2021, corresponding to a percent increase in incidence of 14.3% [95% PI 10.8%–18.0%]. In the scenario combining faster baseline transmission with our local estimate for the relative growth rate of B.1.1.7, the variant would be expected to increase incidence by 199 [95% PI 93–371] cases during this period, corresponding to a percent increase in incidence of 6.2% [95% PI 3.7%–8.4%]. Assuming slower baseline transmission and the published estimate for the B.1.1.7 rate, we would expect 171 [95% PI 70–378] more infections, which is a percent increase of 11.2% [95% PI 8.6%–14.9%] in the study period. Finally, in the most optimistic scenario (slower baseline

transmission combined with our local estimate of ~24% higher B.1.1.7 transmissibility), the projections drop to 67 [95% PI 28–165] more infections, which is an expected increase in incidence of 4.8% [95% PI 3.1%–7.0%].

We compare the model projections across all six scenarios considered (faster versus slower baseline spread combined with either ~59%, ~24% or no increase in transmissibility for B.1.1.7) (Appendix Figure 8).

Appendix Figure 9 compares the imputed and projected infections with and without the variant and the total for the faster and slower spread scenarios to show how the B.1.1.7 variant (in red) over time makes up a growing proportion of infections. This is presented in log scale.

## Additional Limitations

In addition to the limitations listed in our letter, we make the following assumptions. First, we assume that PCT testing represents a random sample of the entire student population. However, testing is voluntary. If students are more likely to test after known exposures, then we may overestimate initial prevalence. Alternatively, if students engaging in riskier behaviors are less likely to seek testing, then we may underestimate prevalence.

Second, we assume that SGTF prevalence among positive PCT specimens is representative of SGTF prevalence in the UT Community as whole. However, the location of PCT testing varies each day and is sometimes targeted toward certain populations, and therefore cases tend to cluster geographically by day (*2*). These two factors might increase the chance of detecting a cluster of related B.1.1.7 cases that are not indicative of the overall prevalence of the variant in the UT community. This could lead us to overestimate both its local prevalence and growth rate. However, we note that it is unlikely that B.1.1.7 cases are being systematically selected for testing within the data up to this point. All tests collected before February 5, 2021 at UT occurred before sequencing confirmation of the presence of B.1.1.7 on campus, and no effort was made to perform more aggressive contact-tracing of these individuals before this date.

Third, we note that our indirect estimates of immunity and transmission rates within the UT community are based on limited data from the fall semester of 2020, and thus are highly uncertain.

Fourth, we make projections based on two different estimates for the transmission advantage of the B.1.1.7. Our local estimate based on SGTF data are considerably lower than a previously published estimate (*6*). The discrepancy between our local estimate and prior estimates from other regions of the U.S. and UK (*6*,*13*) may be caused, in part, by the co-circulation of other variants of concern, including the P.1 and B.1.429 variants (*14*,*15*). The presence of highly transmissible variants among the non-SGTF viruses (in addition to wildtype) would lower the estimated growth rate of B.1.1.7 *relative* to such viruses. Additionally, inclement weather in Texas during February 12–18 (*16*), which disrupted local transmission substantially, may have contributed to the lower estimate of variant transmissibility.

Finally, we make the simplifying assumption that infection by either variant renders an individual immune to reinfection by either variant, despite several reports of COVID-19 reinfections (*17*). While reinfection may become more likely as the virus continues to evolve, scientists believe that past infections provide a reasonable degree (but not full) immunity and that reinfections are not a primary driver of B.1.1.7 transmission (*6*).

## Pairwise Analysis of $C_t$ Values among Non-SGTF and SGTF Positive SARS-CoV-2 Samples

To test the hypothesis that the B.1.1.7 variant's increase in transmissibility may be due to higher viral load of individuals infected with the variant, we examined the distribution of Ct values among non-SGTF and SGTF samples from all SARS-CoV-2 positive tests. The Ct value, or cycle threshold, refers to the number of cycles needed to amplify the 3 target genes (N, S, and ORf1ab) of SARS-COV-2 to detectable levels. A higher Ct value indicates a lower viral load. To ensure that the SGTFs analyzed were due to deletions in viral RNA rather than too little viral presence, we previously restricted our analysis of B.1.1.7 prevalence to samples with mean Ct values over all 3 genes or the remaining genes if S gene was not detected (dashed line, Appendix Figure 8). However, to investigate whether SGTF positives have systematically higher viral loads (lower Ct values) we looked at the Ct value of all available SARS-COV-2 positive samples collected from January 16, 2021 to April 9th, 2021 (Appendix Figure 8). In total, there were 130 SGTF positives and 533 non-SGTF positives. Of the 130 SGTF positives, 100 (76.9%) of them

had Ct values less than 28. Of the 533 non-SGTF positives, 393 (73.7%) of them had Ct values less than 28.

Analyzing all of the SGTF and non-SGTF positives, we find that the median Ct value of non-SGTF positives is 24.2 (95% CI 14.0–33.9), while the median Ct value of the SGTF positives is 24.6 (95% CI 14.6- 34.6). To test for significant differences in these two distributions, we ran a two-sample Kolmogorov-Smirnov test on the Ct values of the non-SGTF and SGTF samples, using the ks.test function in R, giving p = 0.8064. We thus see no statistically significant difference between the two distributions.

We find no evidence within this small dataset that SGTF positive individuals have higher viral loads than individuals with non-SGTF positives. This is in contrast to other studies (*18*), which have demonstrated that B.1.1.7 samples have a higher viral load than previously circulating variants, and hypothesize that this higher viral load could be driving the increase in transmissibility of B.1.1.7. Our findings don't contradict this hypothesis, as the samples collected are not necessarily representative of the entire viral load time-course in the population due to the voluntary nature of UT's PCT testing (i.e., student's might be more likely to seek a test after developing symptoms, potentially biasing samples to later in the viral load trajectory after peak viral load has been reached). Additionally, UT's proactive community testing program uses saliva samples rather than nasopharyngeal. It's possible that saliva samples are not as representative of the viral load in the respiratory tract as nasopharyngeal samples.

## Retrospective Analysis through May 7, 2021

The analysis in this manuscript was completed on April 9, 2021. In August of 2021, we retrospectively compared our projections to data reported between April 9 and the end of the spring semester at UT (Figures S11 and S12).

Our estimates of B.1.1.7 prevalence in late April and early May are noticeably lower than the projections, however the error bars are very large given the low numbers of reported cases leading to large uncertainty (Appendix Figure 11, purple points). The lower relative frequency of B.1.1.7 may have partly stemmed from the cocirculation of other variants (such as P.1 and B.1.429) during this period (*14*,*15*). State-level data were consistent with our projections (orange lines).

When evaluating our projections of infections for the remainder of the semester, we also overestimated the total number of infections in both of the faster spread and slower spread scenarios. Looking back, it is now apparent that the two scenarios we chose in which we assumed that in absence of B.1.1.7 Rt would remain near 1, were not what was observed. This could be in part due to the roll out of vaccines in the Austin community, which began in February and became available to students in April. However, the intention of the projections was to assess whether the B.1.1.7 variant would pose a significant risk to infection rates once it came to dominate, and we were qualitatively correct in predicting in both the faster and slower spread scenarios that it would not have a major impact.

This retrospective validation suggests that our early (February) and updated (April) analysis based on limited quantitative PCR data provided reasonable projections and uncertainty quantification for the ascent of the B.1.1.7 variant. In the case of B.1.1.7, the use of SGTF as a proxy enabled early detection and real-time situational awareness from qRT-PCR data alone; however, for future variants, expanded molecular surveillance capacity will be needed to detect new variants and monitor existing variants in real-time and at the local and national scale.

## References


1. University of Texas at Austin. COVID-19 dashboard. 2020 Aug 6 [cited 2021 Feb 12]. https://coronavirus.utexas.edu/ut-austin-covid-19-dashboard

2. University of Texas at Austin. University of Texas Proactive Community Testing Program for COVID-19 [cited 2021 Feb 9]. https://healthyhorns.utexas.edu/coronavirus_proactive_testing.html

3. Bal A, Destras G, Gaymard A, Stefic K, Marlet J, Eymieux S, et al.; COVID-Diagnosis HCL Study Group. Two-step strategy for the identification of SARS-CoV-2 variant of concern 202012/01 and other variants with spike deletion H69-V70, France, August to December 2020. Euro Surveill. 2021;26:2100008. PubMed https://doi.org/10.2807/1560-7917.ES.2021.26.3.2100008

4. Helix. Update on the Helix, Illumina surveillance program: B.1.1.7 variant of SARS-CoV-2, first identified in the UK, spreads further into the US. 2021 Jan 11 [cited 2021 Feb 4]. https://blog.helix.com/b117-variant-updated-data

5. Goodrich B, Gabry J, Ali I, Brilleman S. rstanarm: Bayesian applied regression modeling via Stan. R package version [cited 2021 Feb 4]. https://cran.r-project.org/web/packages/rstanarm/index.html

6. Davies NG, Abbott S, Barnard RC, Jarvis CI, Kucharski AJ, Munday JD, et al.; CMMID COVID-19 Working Group; COVID-19 Genomics UK (COG-UK) Consortium. Estimated transmissibility and impact of SARS-CoV-2 lineage B.1.1.7 in England. Science. 2021;372:eabg3055. PubMed https://doi.org/10.1126/science.abg3055

7. Chevin L-M. On measuring selection in experimental evolution. Biol Lett. 2011;7:210–3. PubMed https://doi.org/10.1098/rsbl.2010.0580

8. Wallinga J, Lipsitch M. How generation intervals shape the relationship between growth rates and reproductive numbers. Proc Biol Sci. 2007;274:599–604. PubMed https://doi.org/10.1098/rspb.2006.3754

9. Griffin J, Casey M, Collins Á, Hunt K, McEvoy D, Byrne A, et al. Rapid review of available evidence on the serial interval and generation time of COVID-19. BMJ Open. 2020;10:e040263. PubMed https://doi.org/10.1136/bmjopen-2020-040263

10. Cori A, Ferguson NM, Fraser C, Cauchemez S. A new framework and software to estimate time-varying reproduction numbers during epidemics. Am J Epidemiol. 2013;178:1505–12. PubMed https://doi.org/10.1093/aje/kwt133

11. Volz E, Mishra S, Chand M, Barrett JC, Johnson R, Geidelberg L, et al.; COVID-19 Genomics UK (COG-UK) consortium. Assessing transmissibility of SARS-CoV-2 lineage B.1.1.7 in England. Nature. 2021;593:266–9. PubMed https://doi.org/10.1038/s41586-021-03470-x

12. Matsui C, Johnson K, Pasco R, Lachmann M, Fox SJ, Ancel Meyers L. COVID-19 campus introductions and gathering risks for reopening the University of Texas at Austin. 2020 Aug 20 [cited 2021 Feb 4]. https://sites.cns.utexas.edu/sites/default/files/cid/files/ut_reopening.pdf?m=1598027935

13. Washington NL, Gangavarapu K, Zeller M, Bolze A, Cirulli ET, Schiabor Barrett KM, et al. Emergence and rapid transmission of SARS-CoV-2 B.1.1.7 in the United States. Cell. 2021;184:2587–2594.e7. PubMed https://doi.org/10.1016/j.cell.2021.03.052

14. Morales L. Highly transmissible coronavirus variant first found in California detected on UT campus. Austin American Statesman. 2021 Mar 16 [cited 2021 Apr 16]. https://www.statesman.com/story/news/2021/03/16/highly-transmissible-coronavirus-variant-first-found-california-detected-ut-campus/4712790001

15. University of Texas at Austin. Protect Texas together: announcements. 2020 Apr 16 [cited 2021 Apr 18]. https://coronavirus.utexas.edu/campus-announcements

16. Ferman M, Limón E, Sparber S. 2 million Texas households without power as massive winter storm drives demand for electricity. Texas Tribune. 2021 Feb 15 [cited 2021 Apr 18]. https://www.texastribune.org/2021/02/15/rolling-blackouts-texas

17. Centers for Disease Control and Prevention. Reinfection with COVID-19. 2021 Jan 27 [cited 2021 Feb 12]. https://www.cdc.gov/coronavirus/2019-ncov/your-health/reinfection.html

18. Calistri P, Amato L, Puglia I, Cito F, Di Giuseppe A, Danzetta ML, et al. Infection sustained by lineage B.1.1.7 of SARS-CoV-2 is characterised by longer persistence and higher viral RNA loads in nasopharyngeal swabs. Int J Infect Dis. 2021;105:753–5. PubMed https://doi.org/10.1016/j.ijid.2021.03.005

19. Matsui C, Johnson KE, Pasco R, Lachmann M, Fox SJ, Meyers LA. COVID-19 campus introduction risks for spring 2021 at the University of Texas at Austin. 2021 Jan 29 [cited 2021 Feb 4]. https://sites.cns.utexas.edu/sites/default/files/cid/files/ut_spring_introductions.pdf?m=1611961704

20. Backer JA, Klinkenberg D, Wallinga J. Incubation period of 2019 novel coronavirus (2019-nCoV) infections among travellers from Wuhan, China, 20–28 January 2020. Euro Surveill. 2020;25(5). PubMed https://doi.org/10.2807/1560-7917.ES.2020.25.5.2000062

21. Walsh KA, Spillane S, Comber L, Cardwell K, Harrington P, Connell J, et al. The duration of infectiousness of individuals infected with SARS-CoV-2. J Infect. 2020;81:847–56. PubMed https://doi.org/10.1016/j.jinf.2020.10.009

**Appendix Table 1.** Weekly SGTF and total positive samples reported by UT PCT and estimated percent of COVID-19 cases that are infected by the B.1.1.7 variant in the UT community. Estimates are given as posterior medians and 95% credible intervals for the Friday of the week indicated. Bold lines separate contemporaneous estimates from projections, with italics indicating future projections based on observed data through either February 12 or April 9, 2021

| Period | Samples with SGTF | Total COVID-19 positive samples | Estimated percent of cases infected by B.1.1.7 variant using data through Feb 12 | Estimated percent of cases infected by B.1.1.7 variant using data through Apr 9 |
|---|---|---|---|---|
| Jan 16–22 | 1 | 49 | 4.2% [1.7%–8.6%] | 8.3% [5.3%–12.5%] |
| Jan 23–29 | 5 | 93 | 6.9% [4.0%–10.6%] | 10.5% [7.2%–14.8%] |
| Jan 30–Feb 5 | 15 | 75 | 11.2% [7.5%–15.9%] | 13.3% [9.6%–17.6%] |
| Feb 6–12 | 11 | 83 | 17.9% [9.6%–29.4%] | 16.5% [12.6%–20.9%] |
| Feb 13–19† | 0 | 0 | 27.3% [11.2%–51.2%] | 22.3% [14.2%–21.8%] |
| Feb 20–26 | 23 | 55 | 39.1% [12.7%–73.3%] | 24.9% [20.4%–29.9%] |
| Feb 27–Mar 5 | 3 | 24 | 52.3% [14.2%–87.9%] | 30.1% [24.8%–35.8%] |
| Mar 6–12 | 4 | 19 | 65.2% [15.9%–95.1%] | 35.9% [29.4%–42.8%] |
| Mar 13–19 | 2 | 5 | 76.3 [17.6%–98.1%] | 42.0% [34.1%–50.3%] |
| Mar 20–26 | 8 | 31 | 84.6% [19.5%–99.3%] | 48.4% [38.8%–58.1%] |
| Mar 27–Apr 2 | 11 | 16 | 88.0% [20.0%–99.6%] | 54.9% [43.7%–65.7%] |
| Apr 2–Apr 9 | 16 | 21 | 92.5% [21.7%–99.8%] | 61.2% [48.6%–72.3%] |
| Apr 10–Apr 16 | NA | NA | 95.5% [23.8%–99.9%] | 67.2% [53.4%–77.9%] |
| Apr 18–Apr 23 | NA | NA | 97.3% [26.0%–100.0%] | 72.6% [58.8%–84.4%] |
| Apr 24–Apr 30 | NA | NA | 98.4 [28.3%–100.0%] | 77.5% [62.7%–87.8%] |
| May 1–May 7 | NA | NA | 99.0% [30.7%–100.0%] | 81.7% [67.6%–91.3%] |
| May 8–May 14 | NA | NA | 99.4% [33.1%–100.0%] | 85.2% [71.0%–93.3%] |
| May 15- May 21 | NA | NA | 99.7% [35.8%–100.0%] | 88.2% [74.7%–95.2%] |

*Estimated for Friday of the specified week.
†No data was collected this week due to the winter snow storm in the Austin area (*18*).

**Appendix Table 2.** Initial conditions for COVID-19 transmission simulations

| Variable | Value |
|---|---|
| Initial day of simulation | 1/16/2021 |
| Initial proportion infected | 1.2% [95% CI 8.4- 1.6%] are infected |
| Initial proportion immune (percent of students previously infected, as estimated from fall UT testing data) | 30.1% [95% CI 24.9- 36.1%] of UT students are immunized from prior infection (*1*,*10*) |
| Day of projection initialization | 4/9/2021 |
| Prevalence of B.1.1.7 variant on day of projection initialization | 62.7% [95% CI 50.1%–73.9%] of cases are infected by B.1.1.7 |

**Appendix Table 3.** Model parameters

| Parameters | Value | Source |
|---|---|---|
| *N*: Number of UT students in Austin | 30,000 | (*19*) |
| $\gamma$: transition rate from exposed to infectious | 1/3 | (*20*) |
| $\delta$ recovery rate | 1/7 | (*21*) |
| M: relative transmissibility of the variant | Literature: Triangular(1.56, 1.59, 1.63)<br>Locally derived: 1.26 [95% CI 1.19–1.34] | (*6*) |
| Rt: reproduction number | Slower scenario:<br>0.85 [95% CI 0.67–1.07]<br>Faster scenario:<br>1.17 [95% CI 0.82–1.29] | Estimated using EpiEstim (*10*) from UT PCT data (*1*) |
| $\beta(t)$: transmission rate | Slower scenario:<br>0.25 [95% CI 0.18, 0.36]<br>Faster scenario:<br>0.35 [95% CI 0.26, 0.52] | Calculated from Rt |

**Appendix Table 4.** Scenario parameters

| Transmission scenarios after April 9th | |
|---|---|
| All 6 combinations of these two factors | The wildtype spreads either slower ($R_t$ = 0.85 [95% CI 0.67–1.07]) or faster ($R_t$ = 1.17 [95% CI 0.82–1.29])<br>The B.1.1.7 variant either:<br>1. Has higher transmissibility, as reported in (*6*) (M = 1.59 [95% CI 1.56- 1.63])<br>2. Has higher transmissibility, as estimated from local data (M = 1.24 [95% CI 1.17–1.32])<br>Does not have higher transmissibility |

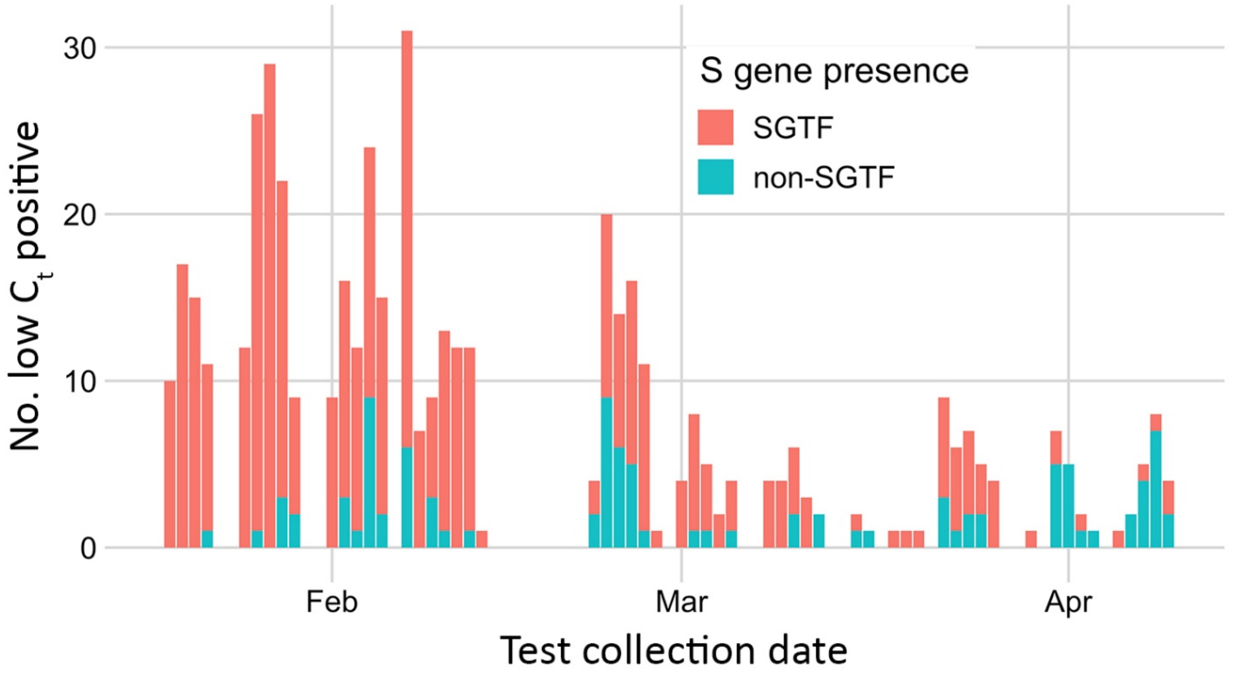


**Appendix Figure 1.** Daily numbers of positive SARS-CoV-2 samples with Ct<28 reported by UT PCT from January 16 to April 9, 2021, stratified by non-SGTF (blue) and SGTF (red).

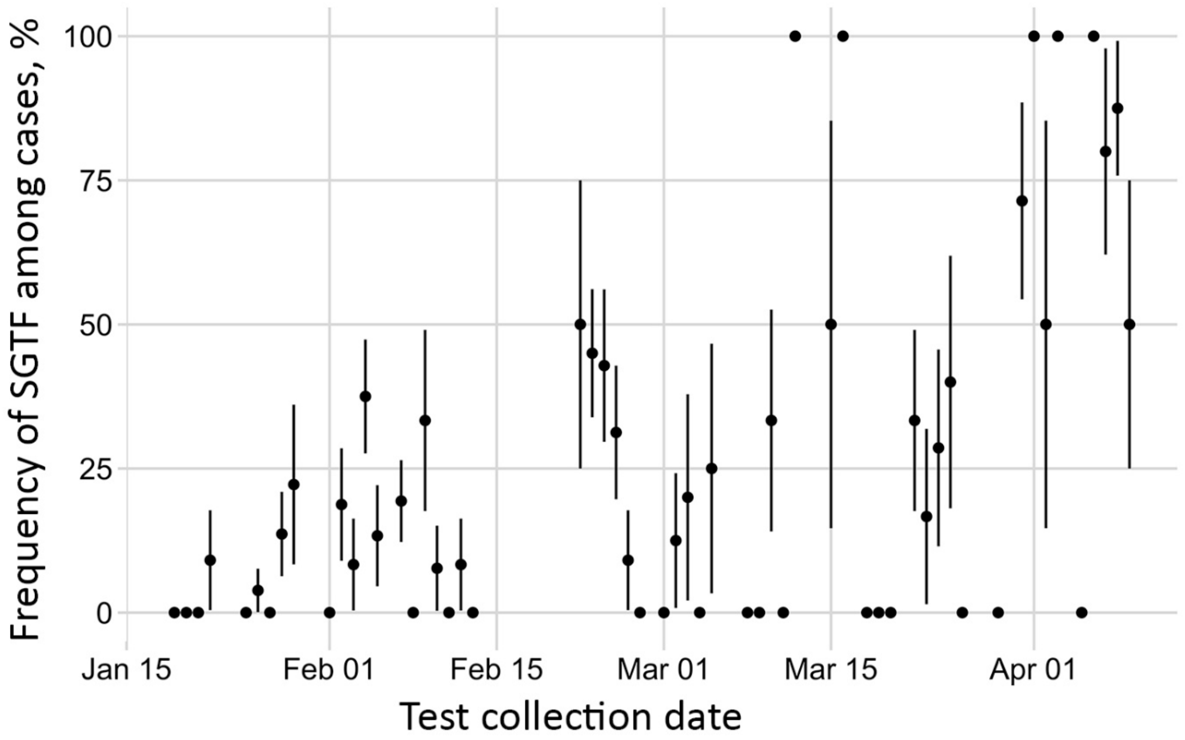


**Appendix Figure 2.** Daily frequency of SGTF among positive SARS-CoV-2 samples with Ct<28 reported by UT PCT from January 16 to April 19, 2021. Vertical lines indicate standard error.

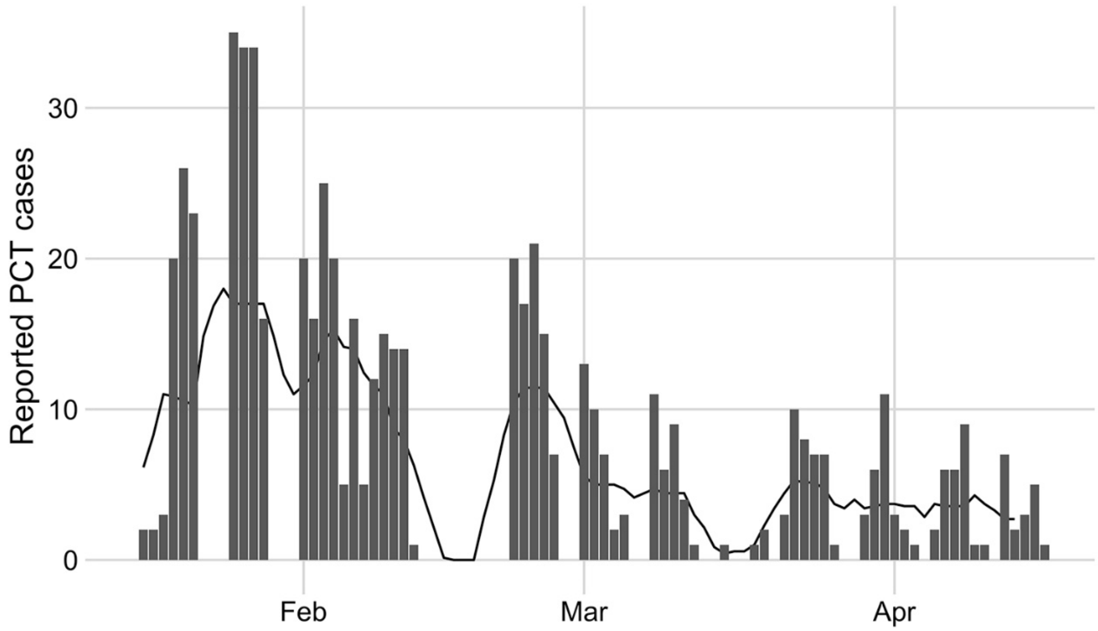


**Appendix Figure 3.** SARS-CoV-2 infections among UT students reported by the PCT testing program. Bars indicate the daily reported positive cases. The trend line represents the 7-day rolling average.

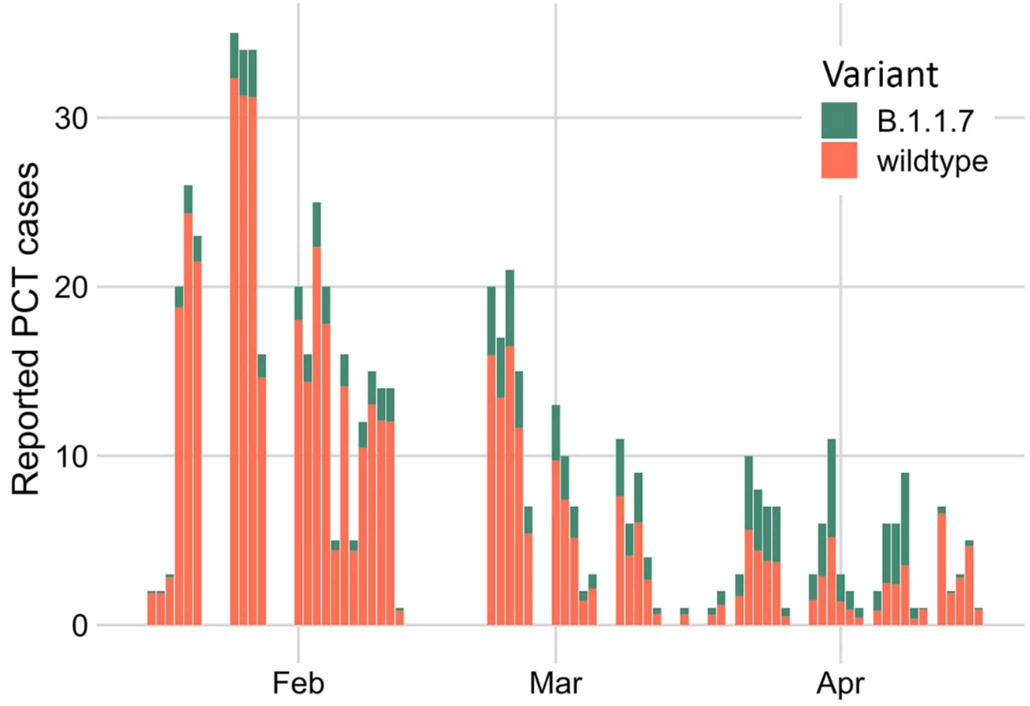


**Appendix Figure 4.** SARS-CoV-2 infections among UT students reported by the PCT testing program, classified as B.1.1.7 or wildtype, based on our median estimates of B.1.1.7 prevalence over time.

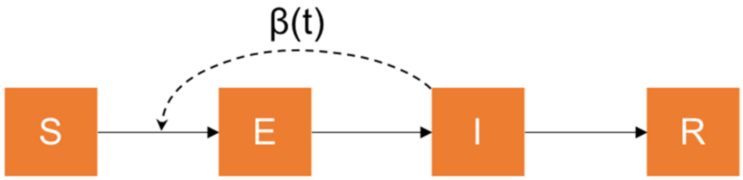


**Appendix Figure 5.** Diagram of the COVID-19 transmission model. Upon exposure, susceptible individuals (S) progress to exposed, from which they move to the infected compartment. The transmissibility of infected individuals is a function of the proportion of cases that are B.1.1.7 and the baseline transmission rate. All infected individuals progress to the recovered state where they remain protected from future infection (R).

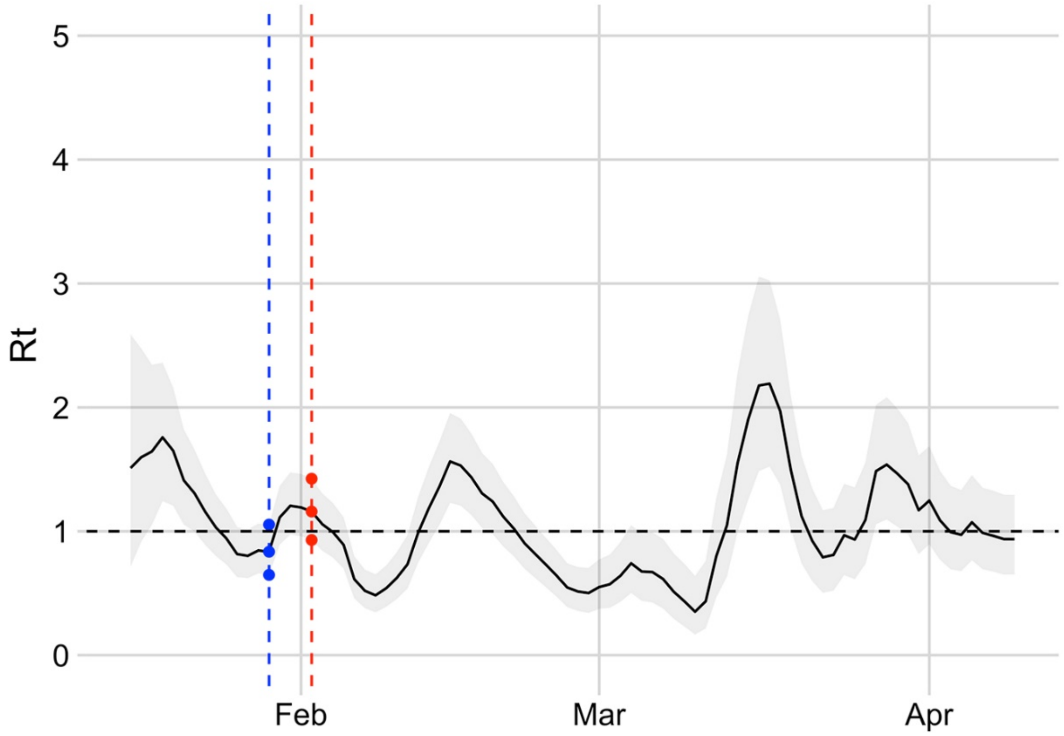


**Appendix Figure 6.** Effective reproductive number, $R_t$, from January 16 to April 9th, 2021. The black solid line and gray shading indicate the median and 95% CI estimated $R_t$, respectively. The horizontal dashed line is the epidemic threshold of $R_t = 1$. The blue dots and vertical line on January 29, 2021 represent the $R_t$ value of 0.85 [95% CI 0.67–1.07] used in the “slower spread” scenario. The red dots and vertical line on February 2, 2021 represent the $R_t$ value of 1.17 [95% CI 0.82- 1.29] used in the “faster spread” scenario.

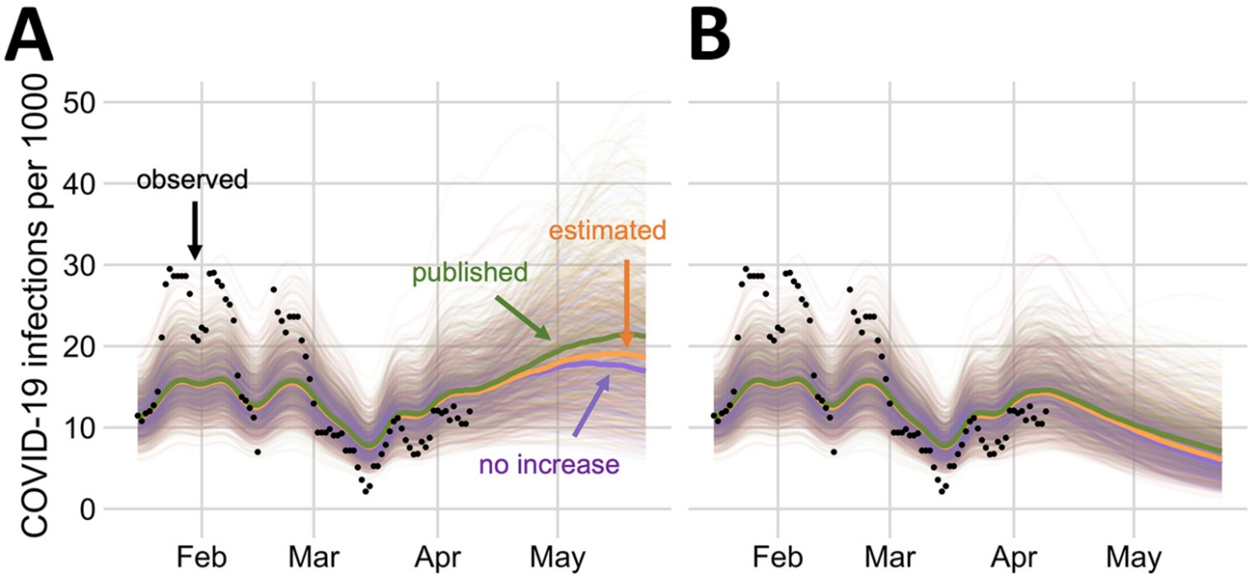


**Appendix Figure 7.** Projected COVID-19 cases at UT throughout the spring semester of 2021 under four transmission and variant scenarios. In both graphs, green lines correspond to a scenario in which B.1.1.7 has ~59% higher transmissibility (*6*), orange lines assume a local estimate of ~24% higher transmissibility, and purple lines assume no increase in transmissibility. The black dots indicate observed cases detected through UT Proactive Community Testing (PCT) per 1000 (seven-day average). The left graph shows projections under a faster transmission scenario ($R_t$ = 1.17 [95% CI 0.82–1.29]) and the right graph assumes a slower transmission scenario ($R_t$ = 0.85 [95% CI 0.67–1.07]). For each scenario, we display 500 simulations, with the bold line indicating the median projected value on each day.

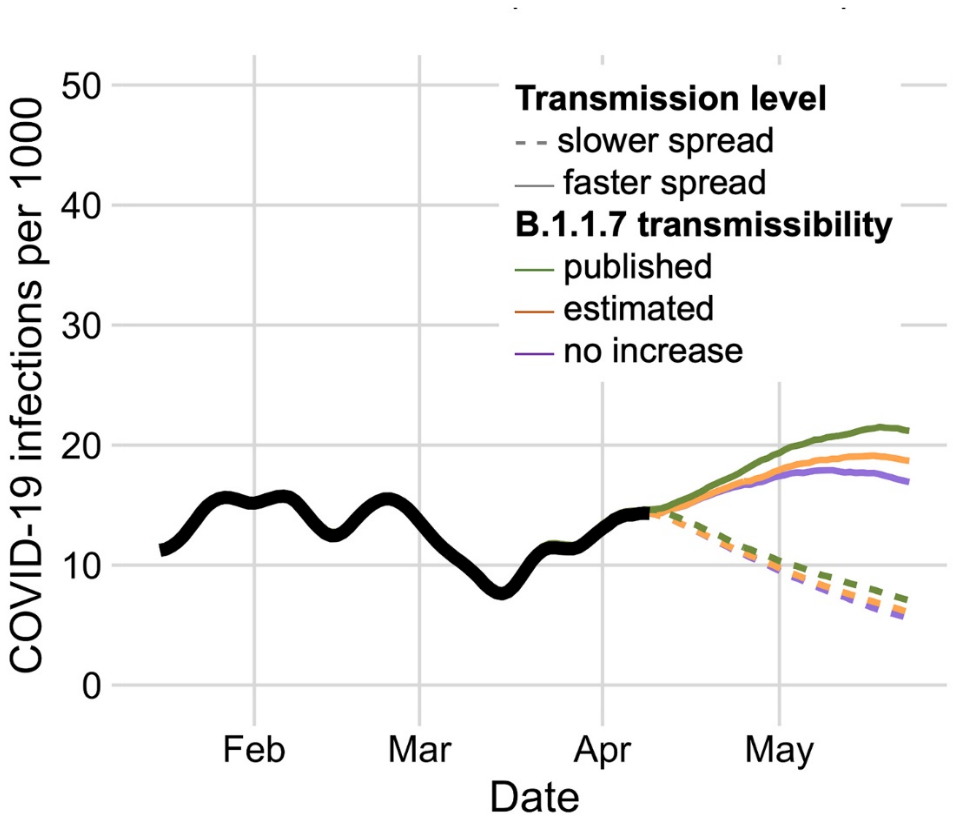


**Appendix Figure 8.** Median imputed and projected SARS-CoV-2 incidence by scenario. Slower and faster baseline spread is indicated by dashed and solid lines, respectively. Colors indicate the assumed relative transmission rate of B.1.1.7, with green, orange and purple corresponding to large (~59%), moderate (24%), and no increase relative to the wildtype, respectively. Black solid line corresponds to the median imputed COVID-19 infections per 1000 estimated using data up through April 9th, 2021.

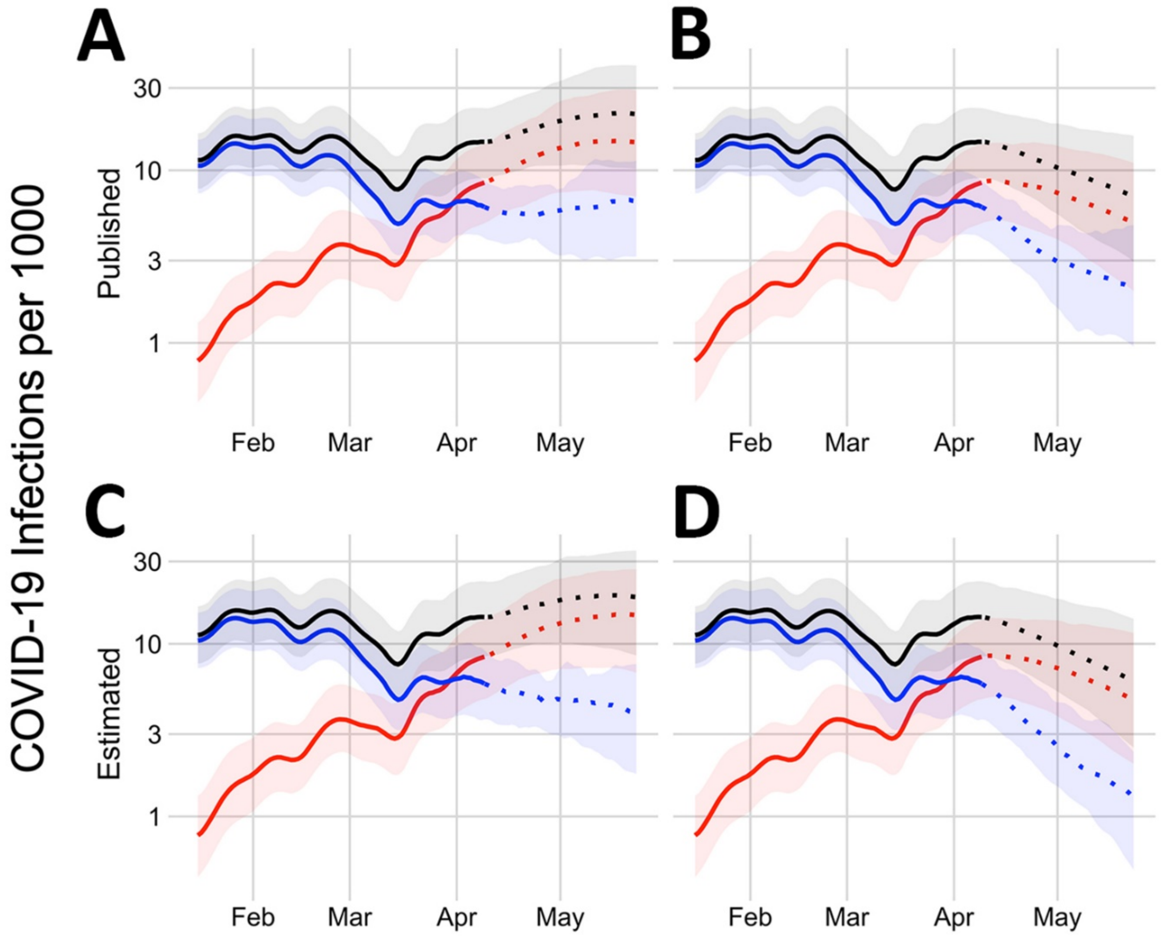


**Appendix Figure 9.** Imputed (solid lines) and projected (dotted lines) total infections (black), wildtype infections (blue), and B.1.1.7 infections (red) under the faster spread scenario (left) and slower spread scenario (right). The top graphs assume a local estimate for the transmissibility of B.1.1.7 (~24% more transmissible than the wildtype) and the bottom graphs assume a published estimate (~59% more transmissible). The left column is for the faster transmission scenario ($R_t$ = 1.17 [95% CI 0.82–1.29]) and the right column is for the slower transmission scenario ($R_t$ = 0.85 [95% CI 0.67–1.07]). Solid lines indicate imputed infections, dotted lines indicate projected infections. Incidence estimates/projections are presented on a log scale.

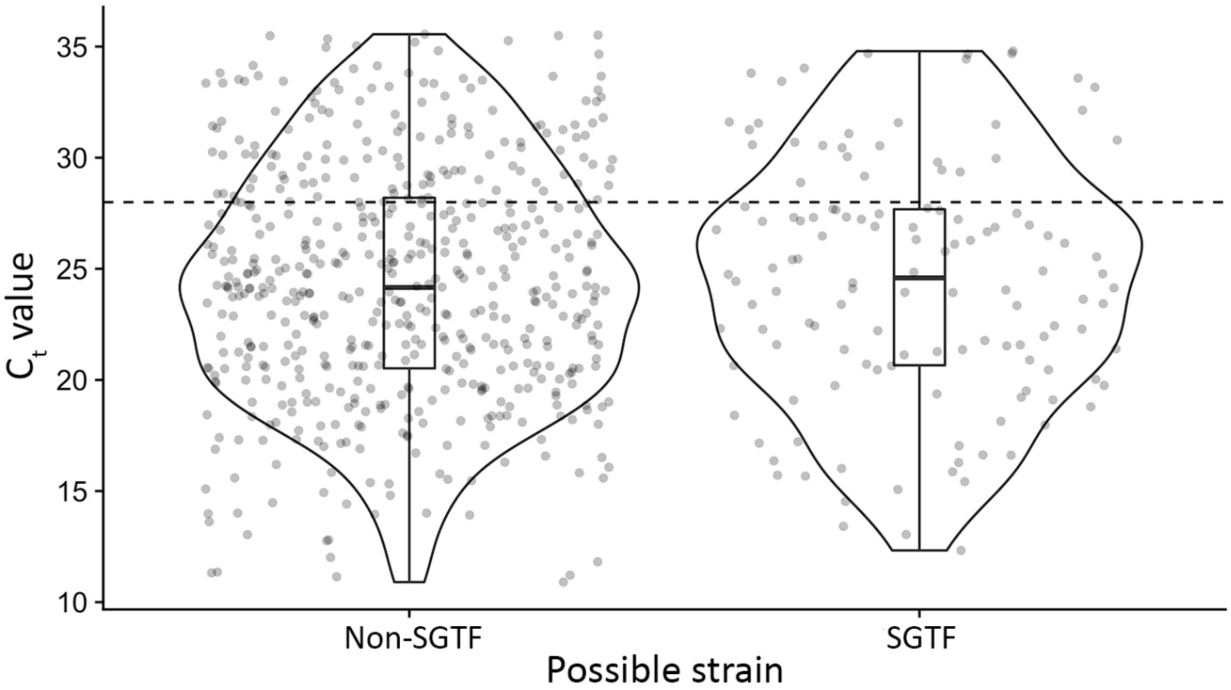


**Appendix Figure 10.** Ct values of non-SGTF versus SGTF positive SARS-COV-2 samples collected from PCT from January 16, 2021 to April 9, 2021. Dashed line at Ct = 28 indicates the threshold value below which positive SARS-CoV-2 samples were included in the estimates of B.1.1.7 prevalence. Box plots indicate the median and quartiles over all Ct values for each group. Dots indicate individual sample Ct values. Violin plots show density of samples at each Ct value.

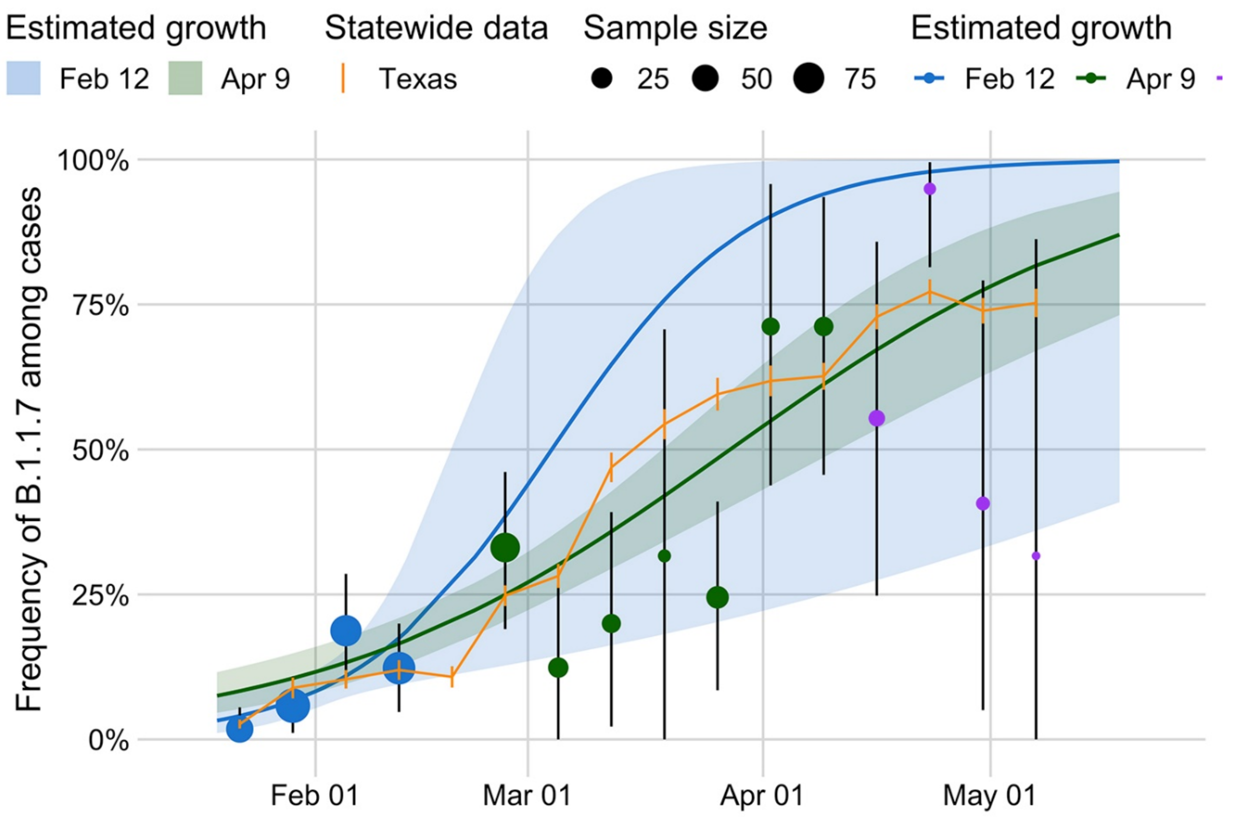


**Appendix Figure 11.** Estimated frequency of the B.1.1.7 variant among positive COVID-19 cases at the University of Texas, January 16 - May 7, 2021. Based on the number of samples with SGTF among SARS-CoV-2 positive samples reported by UT Proactive Community Testing (PCT), we estimate the weekly frequency of the B.1.1.7 variant (points), with vertical error bars indicating 95% confidence intervals. We fit a logistic growth model to data through February 12 (blue) and April 9 (green) to project the prevalence of the B.1.1.7 variant relative to the previously circulating (wildtype) virus through May 23. Shaded bands indicate 95% credible intervals, which reflect uncertainty in the percent of cases that are S gene dropouts, the percent of S gene dropouts that are B.1.1.7, and the fitted model parameters. The 95% credible interval of our initial projections (blue shading) contains the posterior median estimated from subsequent data (green line). The purple points provide estimates of B.1.1.7 prevalence, based on data from April 9 through the end of the spring semester (May 7). The orange line indicates B.1.1.17 prevalence among sequences submitted to GISAID from the state of Texas.

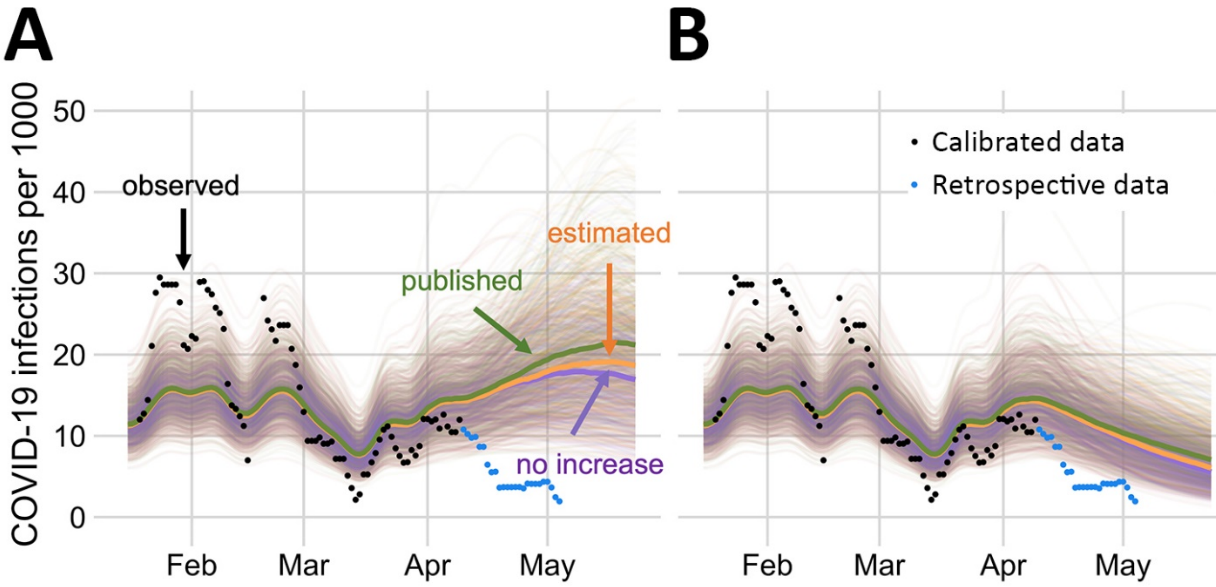


**Appendix Figure 12.** Projected COVID-19 cases at UT throughout the spring semester of 2021 under four transmission and variant scenarios. In both graphs, green lines correspond to a scenario in which B.1.1.7 has ~59% higher transmissibility (*6*), orange lines assume a local estimate of ~24% higher transmissibility, and purple lines assume no increase in transmissibility. The black dots indicate observed cases detected through UT Proactive Community Testing (PCT) per 1000 (seven-day average). The left graph shows projections under a faster transmission scenario ($R_t$ = 1.17 [95% CI 0.82–1.29]) and the right graph assumes a slower transmission scenario ($R_t$ = 0.85 [95% CI 0.67–1.07]). For each scenario, we display 500 simulations, with the bold line indicating the median projected value on each day. The blue points represent the data collected after these projections were calculated, between April 9 and May 7, 2021.
